# Sex-Biased Immune Responses to Antibiotics during Anti-PD-L1 Treatment in Mice with Colon Cancer

**DOI:** 10.1155/2022/9202491

**Published:** 2022-07-19

**Authors:** Nan Jing, Luoyang Wang, Huiren Zhuang, Chao Ai, Guoqiang Jiang, Zheng Liu

**Affiliations:** ^1^Department of Chemical Engineering, Tsinghua University, 100084 Beijing, China; ^2^Key Lab of Industrial Biocatalysis, Ministry of Education, Tsinghua University, 100084 Beijing, China; ^3^School of Basic Medicine, Qingdao University, 266071 Qingdao, China; ^4^Beijing Tsinghua Changgung Hospital, 102218 Beijing, China

## Abstract

Colitis is a frequently occurred side effect of immune checkpoint inhibitors (ICIs), which are increasingly used in cancer treatment, whereas antibiotics are widely used to treat colitis, their effectiveness in ICI-associated colitis remains controversial. In this study, we firstly assessed the effectiveness of several commonly used antibiotics and antibiotic cocktails in alleviating of dextran sulfate sodium- (DSS-) induced colitis. We observed that two narrow-spectrum antibiotics, neomycin and metronidazole, were more effective in alleviating colitis, as evidenced by the remission of loss of the body weight, enlargement of the spleen, shortening of the colon, secretion of proinflammatory cytokines, and histological score of the colon tissue. Moreover, these two antibiotics resulted in better relief of colitis symptoms in the MC38 tumor-bearing male mice receiving the anti-PD-L1 mAb (*α*PD-L1) treatment, compared to the females. In the meantime, an enhanced response to *α*PD-L1 efficiency against mice colon cancer was observed in the male mouse group upon the application of these two antibiotics. In contrast, both neomycin and metronidazole showed destructive effects on the antitumor efficiency of *α*PD-L1 in female mice, despite relief from colitis. We found that antibiotic treatment attenuated the increased infiltration of granulocytes and myeloid cells in colon tissue induced by DSS in female mice, while reducing the proportion of Th17 cells in male mice. These differences were further associated with the sex-biased differences in the gut microbiota. These findings indicated that sex-dependent alterations in the gut microbiota should be considered when applying antibiotics for the treatment of ICI-associated colitis.

## 1. Introduction

In recent years, growing efforts have been made to explore immunotherapy using immune checkpoint inhibitors (ICIs) targeting programmed cell death receptor 1/ligand 1 (PD-1/PD-L1) or cytotoxic T lymphocyte-associated antigen 4 (CTLA-4) [1, 2]. However, their clinical applications are limited by either relatively low response rates or potentially severe adverse events [3]. The response rates can be improved by identifying predictive biomarkers [4] or using them in combination with other anticancer agents [5, 6]. Among immune-related adverse events, colitis is one of the most frequently occurring adverse events during immunotherapy with ICIs [7]. Immunosuppressive therapy, including corticosteroids, biologic agents directly inhibiting cytokines and integrins (*α*4*β*7 integrin), and cytotoxic agents (cyclophosphamide) [8], is applied in the treatment of ICI-associated colitis. However, high-dose corticosteroids are associated with a high risk of infectious complications and metabolic disturbances and may inhibit the efficacy of antitumor therapy using anti PD-1 [9]. Targeted cytokine blocking therapies, such as infliximab for TNF-*α* and tocilizumab for IL-6, are specific to a single inflammatory factor, which may also reverse the antitumor effects of ICIs [10, 11]. Fecal microbiota transplantation (FMT) is proven to be effective in the treatment of ICI-associated colitis; however, the safety of this method needs to be examined in a larger cohort [12].

Antibiotics such as penicillin [13], tetracyclines [14], and antibiotic cocktail [15] have been extensively tested for the treatment of colitis. Jin et al. [13] reported that low-dose penicillin inhibited the expression of IL-17, reduced the number of pathogenic Th17 cells in the intestinal tissue, and relieved dextran sulfate sodium- (DSS-) induced colitis. Garrido-Mesa and colleagues [14] found that tetracycline inhibited DSS-induced colitis by enhancing the protective immune pathway, facilitating the healing of the colon mucosa. Rakoff-Nahoum et al. [15] showed that an antibiotic cocktail (ampicillin+vancomycin [Van]+neomycin [Neo]+metronidazole [Met]) leads to reduced survival, significant weight loss, and severe fecal congestion in DSS-induced colitis. Derosa et al. found that application of broad-spectrum antibiotics (quinolones+macrolides+sulfonamides+tetracyclines) led to undesirable changes to the intestinal microbiota [16, 17] and thus had an adverse influence on ICI therapy. Routy et al. revealed the connection between broad-spectrum antibiotic consumption and a poor response to the treatment of epithelial tumors with PD-1 blockade [18]. They also showed that dysbiosis caused by broad-spectrum antibiotics (ampicillin+colistin+streptomycin) significantly compromised the primary resistance to PD-1 blockade in MCA-205 tumor-bearing mice [18]. Growing efforts have been directed toward understanding the relationship between the antibiotic administration and ICI therapy. Pederzoli et al. found that antibiotics taken during PD-1 blockade therapy were associated with lower rates of complete response and recurrence-free survival among patients with muscle-invasive bladder cancer [19]. Hopkins et al. reported that antibiotic use was associated with worse survival outcomes to PD-L1 blockade therapy in urothelial carcinoma patients [20]. In contrast to these conclusions, Cortellini and colleagues revealed that prior antibiotic therapy does not impair clinical outcomes in patients with non-small-cell lung cancer (NSCLC) [21]. Specifically, Lurienne et al. found that exposure shortly before or after ICI initiation seems to be detrimental, whereas later use of antibiotics during the disease course does not alter the survival rate [22]. Recently, Quarni et al. reported that the combined use of anti-PD-L1 mAb (*α*PD-L1) with Mit-A, a polyketide antibiotic capable of inhibiting the binding of the transcription factor SP1 to the minor groove of DNA [23], decreased tumor growth in MC38 tumor-bearing mice [24]. Our previous study showed that a narrow-spectrum antibiotic, colistin, strengthened the immunotherapy efficiency of anti-PD-L1 in male mice but not in female mice [25]. Given all these findings, we came to the present study aimed to probe the interplay of antibiotics, gut microbiota, and colitis in the context of ICI therapy, which we believe may shed light on the understanding of the above-mentioned complicated responses and, moreover, the application of immunotherapy with ICIs.

The present study examined the effectiveness of narrow-spectrum antibiotics and an antibiotic cocktail in the treatment of DSS-induced colitis model. Neomycin and metronidazole, two antibiotics with superior anti-inflammatory effects, were tested in the treatment of MC38 tumor-bearing mice with *α*PD-L1. While both antibiotics demonstrated excellent anti-inflammatory effects, a sexual dimorphism in the antitumor efficiency of *α*PD-L1 appeared. Moreover, the gut microbiota and immune systems, indexed by the type and content of immune cells in male and female mice, responded distinctively to these antibiotics. These findings are essential for the application of narrow-spectrum antibiotics for the treatment of colitis associated with ICI therapy.

## 2. Materials and Methods

### 2.1. Animals and Antibiotic Treatment

Seven-week-old male and female C57BL/6 mice (Vital River Laboratory Animal Technology Co. Ltd, Beijing, China) were housed under pathogen-free conditions at the Laboratory Animal Resources Center, Tsinghua University, according to approved protocols (No. 20-LZ1-1).

A mouse model of ulcerative colitis was established by oral administration of 2% dextran sulfate sodium (DSS, molecular weight 36000–50000 Da, MP Biomedicals, Irvine, CA) in drinking water for 1 week after 1 week of antibiotic uptake. Antibiotic administration was continued during DSS treatment. For the present study, neomycin (Neo), metronidazole (Met), and vancomycin (Van) were supplied in drinking water at concentrations of 1 g/L, 1 g/L, and 0.5 g/L, respectively [15]. All antibiotics were purchased from Sigma-Aldrich (St. Louis, MO). The antibiotic cocktail group (Abx) was treated with 1 g/L ampicillin, 1 g/L neomycin, 1 g/L metronidazole, 0.5 g/L, and vancomycin. To maintain the concentration of antibiotics and DSS against degradation, these reagents were renewed every 2 days.

Each mouse received 5 × 10^5^ MC38 cells (subcutaneous (s.c.)) on the right flank to establish a colon cancer model after 1 week of adaptation. Dulbecco's modified Eagle medium (DMEM) supplemented with 10% fetal bovine serum and 1% penicillin-streptomycin was used to culture MC38 cells (Cell Bank of the Type Culture Collection, Chinese Academy of Sciences) at 37°C in a 5% CO_2_ atmosphere [26]. Tumor volumes were measured twice a week using the following formula: (width^2^ × length)/2. One week after tumor inoculation, the mice were randomly divided into different groups according to tumor size (*n* = 6) to ensure the same average volume of each group. For the groups treated with *α*PD-L1 (P), on days 7 and 10, each mouse was injected with 200 *μ*g of anti-PD-L1 mAb (*α*PD-L1, BE0101; BioXCell, Lebanon, NH) (intraperitoneal (i.p.)). The control group (CT) mice were injected with IgG2b isotype (BE0090, BioXCell) at the same time. For the antibiotic-treated groups, neomycin (PN) and metronidazole (PM) were added to drinking water at a concentration of 1 g/L for 1 week on days 7 to 14. Experiments in which a combination of *α*PD-L1 and antibiotics was administered to mice were conducted according to a previous study with slight adjustment [27]. For the groups treated with *α*PD-L1 and DSS (PDS) (2.0%, *w*/*v*) DSS was dissolved in drinking water for 1 week at days 7 to 14. Mice in the antibiotic treatment group received *α*PD-L1 (2.0%, *w*/*v*) DSS, neomycin (PDSN), or metronidazole (PDSM) at the same time.

### 2.2. Evaluation of DSS Colitis

The body weight, stool consistency, and rectal bleeding state of the mice were determined daily. The disease activity index (DAI) was evaluated as an index of disease activity by the scores of body weight loss (scored as 0, no change; 1, 1–5% loss; 2, 5–10% loss; and 3, 10–20% loss), stool consistency (0, normal; 1, soft but firm; 2, soft; and 3, diarrhea), and fecal blood (0, none; 1–2, blood; and 3, gross bleeding) [28].

### 2.3. Hematoxylin and Eosin (HE) Staining and Histologic Analysis

For histological analysis, colon tissues were fixed in 4% formalin and embedded in paraffin. Paraffin was then cut into sections and stained with hematoxylin and eosin. Histological changes were evaluated as previously described by Rangan et al. [29]. In brief, the histological scoring system used four scores for inflammatory infiltration of mucosa (0, none; 1, infiltrate around crypt basis; 2, infiltrate reaching to submucosae; 3, extensive infiltration to submucosae; and 4, full-layer infiltration), damage of goblet cells (0, normal; 1, partial loss of goblet cells; 2, loss of multiple goblet cells but with normal epithelial structure; 3, loss of goblet cells in large areas; 4, all destroyed goblet cells), damage to crypts (0, none; 1, loss of a third of the crypts; 2,loss of two-thirds of the crypts; 3, only maintain intact epithelium; 4, loss of all crypts and epithelium), and the degree of injury (0, none; 1, mucosa; 2, mucosa and submucosa; 3, muscularis; 4, full layer).

### 2.4. Flow Cytometry Analysis of Immune Cells

Immune infiltration in the colon lamina propria and tumor was analyzed, as previously described [30]. In brief, the tissues (i.e., tumor and colon) were minced and digested with 1 mg/mL collagenase IV and 0.15 mg/mL DNase I in Hank's balanced salt solution at 37°C for 30 min. Next, single cells were obtained by filtering the digested tissues with a 70 *μ*m cell strainer (BD Biosciences, Franklin Lakes, NJ). For the spleen and mesenteric lymph nodes (MLNs), immune cells were obtained by gently grinding with a plunger of a sterile syringe in a cell strainer. Spleen cells were treated with red blood cell lysis buffer (Beijing Solarbio Science & Technology, Beijing, China) on ice for 15 min. Single cells were stained with anti-mouse antibodies (eBioscience, Thermo Fisher Scientific, Waltham, MA) against CD4 (30-F11)-PE/Cy7, CD8a (53-6.7)-BrilliantViolet605, CD45 (GK1.5)-APC/Cy7, CD25 (PC61.5)-APC, CD19 (1D3)-AlexaFluor700, NK1.1 (PK136)-APC, Ly6C (HK1.4)-PE, Ly6G (1A8)-FITC, F4/80 (BM8)-BrilliantViolet421, and CD11b (M1/70)-APC in the dark (4°C for 30 min). After surface staining, the cells were fixed and permeabilized with a Foxp3/Transcription Factor Staining Buffer set (eBioscience) for intracellular staining in the dark (4°C, 30 min) with anti-mouse antibodies (eBioscience), including Foxp3 (150D)-PE, IL-17A (eBio17B7)-PE/Cy5, and INF*γ* (XMG1.2)-APC. Cells were resuspended in the phosphate-buffered saline after washing twice and analyzed on an LSRFortessa flow cytometer (BD Biosciences). All data were analyzed using the FlowJo software (version 10.6.2, TreeStar,, Ashland, OR).

### 2.5. Enzyme-Linked Immunosorbent Analysis (ELISA)

The cytokines determined were IL-6 and TNF-*α*. After euthanasia, blood was collected from the eye socket, and serum was separated by centrifugation (3000 rpm, 10 min) and frozen at -80°C until assayed for cytokine content by commercial ELISA (eBiosciences). Plates were read at 450 nm using a plate reader following the procedure of the manufacturer's instructions.

### 2.6. RNA Extraction and Quantitative Real-Time PCR

Total RNA was extracted from cecum tissues that had been preserved in RNAlater solution (Invitrogen, Carlsbad, CA) using 1 mL of TRIzol Reagent (Invitrogen) as previously described. RNA was further used for cDNA production using the EasyScript All-in-One First-Strand cDNA Synthesis SuperMix for qPCR (Takara, Tokyo, Japan) according to the manufacturer's instructions. Quantitative real-time (RT) PCR assays were performed to detect the effect of treatments on the mRNA expression of the cytokines TNF-*α*, IL-1*β*, IL-17, and intestinal tight junction proteins claudin-2, ZO-1, and occludin using SYBR Green on a CFX96 Touch Real-Time PCR Detection System (Bio-Rad, Hercules, CA). Specific primers for genes are as follows: GAPDH (forward, AGGTCGGTGTGAACGGATTTG; reverse, GGGGTCGTTGATGGCAACA), TNF-*α* (forward, CCCTCACACTCAGATCATCTTCT; reverse, GCTACGACGTGGGCTACAG), and IL-1*β* (forward, GCAACTGTTCCTGAACTCAACT; reverse, ATCTTTTGGGGTCCGTCAACT). All primers were designed in http://www.ncbi.nlm.nih.gov/ and synthesized by Thermo Fisher Scientific (Waltham, MA). The average cellular mRNA expression levels were calculated as previously described [13].

### 2.7. Myeloperoxidase Activity Assay

The activity of intestinal myeloperoxidase (MPO) was determined using an MPO assay kit (Nanjing Jiancheng Bioengineering Institute) [31]. Briefly, after the intestinal tissue was weighed, the homogenate medium was added at a volume ratio of 1 : 19 to prepare a 5% tissue homogenate and kept at 37°C for 15 minutes. Then, 3 mL of the chromogenic agent was added to the sample tube in a water bath at 37°C and incubated for 30 minutes. Finally, the samples were incubated at 60°C for 10 minutes with the addition of 0.05 mL of 3,3′-dimethoxybenzidine (0.17 mg/mL) before absorbance measurement at 460 nm. MPO activity in the supernatant was determined by measuring the H_2_O_2_-dependent oxidation and expressed as units per gram of total protein (U/g wet weight of colon tissue) according to the following formula: MPO activity = (OD_sample_ − OD_control_)/(11.3 × sample weight(g)).

### 2.8. Microbial Analysis by 16S rRNA Gene Sequencing

The colon contents of mice were collected at the end of the experiment to extract genomic DNA using a QIAamp DNA Stool Mini Kit (Qiagen, Hilden, Germany) according to the manufacturer's instructions. DNA amplification and sequencing were performed by Major (Shanghai Major Bio-Pharm Technology, Shanghai, China). The 16S rRNA gene amplification methods targeting the V3–V4 hypervariable region and sequencing analysis were consistent with our previous experiments [32]. Alpha diversity was indicated by different indexes including richness, Shannon, Simpson, Chao1, and ACE, using the “vegan” package in R (R Foundation, Vienna, Austria). Principal component analysis (PCA) was performed using the R package “ade4” for beta-diversity. Statistical analysis of the taxonomic profiles was performed using STAMP v2.1.3, and statistically significant differences were calculated using one-way analysis of variance (ANOVA). Redundancy analysis (RDA) was performed using the “vegan” R package, with normalized operational taxonomic unit (OUT) abundance and environmental factors [33]. Linear discriminant analysis (LDA) effect size (LEfSe, http://huttenhower.sph.harvard.edu/galaxy) was used to identify the discriminating OTUs. The LDA threshold value was set to 2.0. Differentially abundant OTUs were used to calculate the microbial dysbiosis index (MDI) [34], using the following formula: log (total abundance of OTUs increased after DSS treatment)/(total abundance of OTUs decreased after DSS treatment). BugBase analysis was performed online (http://bugbase.cs.umn.edu) to obtain phenotypic information based on 16S rRNA gene sequences.

### 2.9. Statistical Analysis

GraphPad Prism 8.0 (GraphPad Software, San Diego, CA) was used for all analyses and data are presented as mean ± standard error of the mean (SEM). Student's *t* test was used to analyze pairwise differences and one-way ANOVA corrected for multiple comparison.

## 3. Results

### 3.1. Alleviating DSS-Induced Colitis in Female Mice with Different Narrow-Spectrum Antibiotics

**Figure 1:**
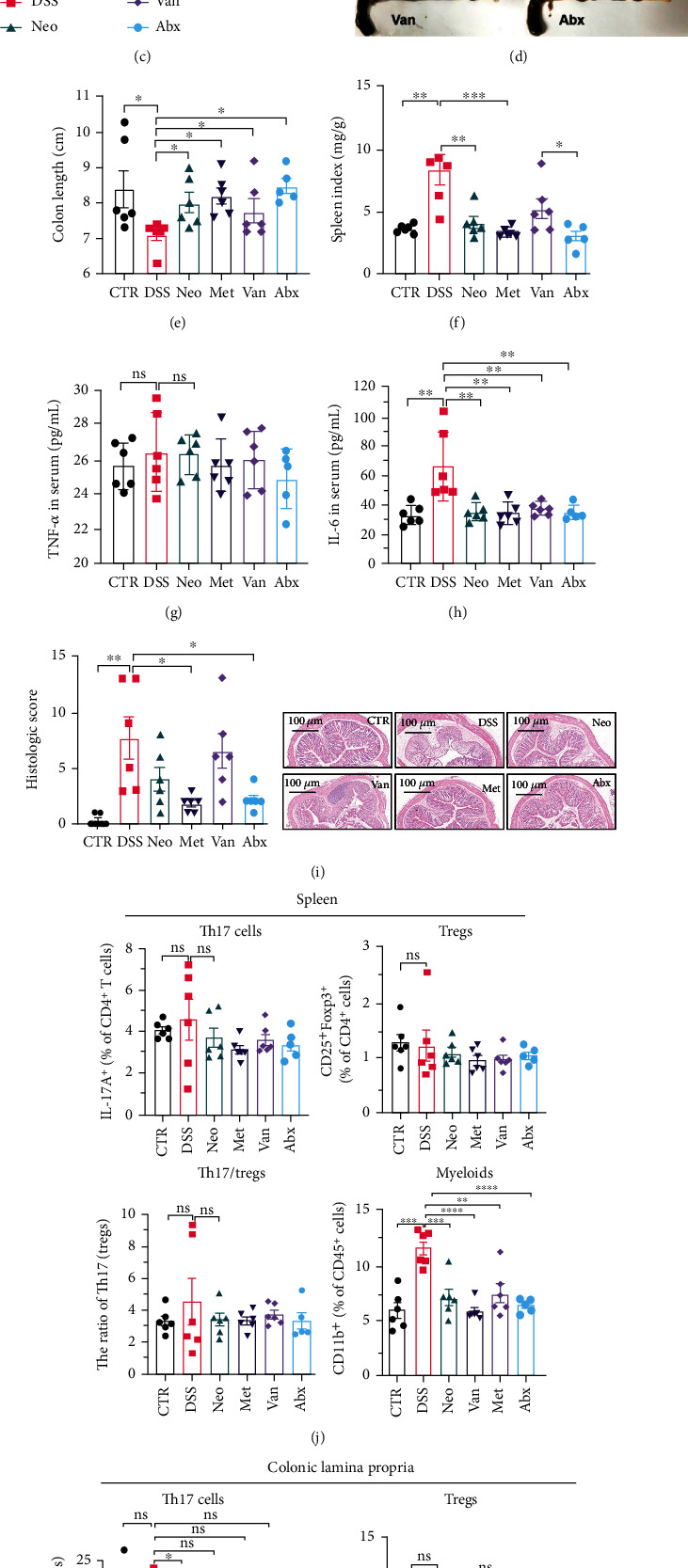
Effects of different antibiotics on DSS-induced colitis in female mice. (a) Study design: 6-week female C57BL/6 mice were given vancomycin, metronidazole, neomycin, antibiotic cocktail (mixture of vancomycin, metronidazole, neomycin, and ampicillin), or regular water as control, respectively, for one week, and then challenged with 2% DSS (*n* = 6/group). (b) Body weight variation during the whole experiment process. (c) DAI score after DSS treatment. (d) Representative images and colon length (e) of the mouse colon. (f) Spleen index of different groups. Quantification of TNF-*α* (g) and IL-6 (h) in serum. (i) Histologic score and representative imaging of hematoxylin and eosin staining. (j) FACS analysis of immune cells in spleen including Th17 cells, Tregs, and myeloids. (k) FACS analysis of immune cell infiltration in colonic lamina propria including Th17 cells, Tregs, and myeloid cells. All data were presented as means ± SEM. ^∗^*P* < 0.05,  ^∗∗^*P* < 0.01, and^∗∗∗^*P* < 0.001.

Before DSS treatment, mice were exposed to Neo, Met, Van, and an antibiotic cocktail consisting of ampicillin, Neo, Met, and Van, respectively, in drinking water for one week, followed by DSS treatment for another week, while antibiotics continued to be administered (Figure 1(a)). The degree of splenic enlargement was calculated by the spleen index with the following formula: splenic weight (mg)/total body weight (g). As shown in Figure 1, antibiotic administration exhibited a protective effect on the decline of body weight (Figure 1(b)). The DAI score in the antibiotic-treated groups was much lower than that in the DSS group (Figure 1(c)), and the narrow-spectrum antibiotics were more effective than Abx, especially in the Neo group. The application of Neo and Met relieved the shortening and hyperemia of the colon in the DSS group (Figures 1(d) and 1(e)), whereas the Van and Abx groups showed severe fecal occult blood, and the cecum of these mice increased markedly (Figure 1(d)). In addition, antibiotic administration significantly alleviated splenic enlargement (Figure 1(f)). We also determined the serum levels of IL-6 and TNF-*α*. As shown in Figure 1(g), there was no significant difference in the levels of TNF-*α* among the groups, except DSS-treated mice that had slightly higher levels of TNF-*α*. However, IL-6 expression increased remarkably in DSS-treated mice (Figure 1(h)). All antibiotic-treated mice showed significantly alleviated elevation of IL-6 induced by DSS. Histological analysis further revealed differences in morphological damage of the colon among the groups (Figure 1(i)). Variable lymphocytic infiltration and crypt destruction of the epithelium were observed in all groups. Notably, DSS-treated mice displayed more severe crypt epithelial cell dropout and destruction, while the Met and Abx groups showed the least damage to colon tissue. The variation in immune cells in the spleen and colonic lamina propria was detected by fluorescence-activated cell sorting using the gating strategy detailed in Supplementary Figure S1. The proportions of CD45^+^ and CD4^+^ T cells in the spleen decreased in the DSS group, whereas antibiotic treatment upregulated it to normal levels (Figure S2A). As shown in Figure 1(j), there was no significant difference in the proportion of Th17 and regulatory T cells (Tregs) in the spleen among all groups. However, the ratio of CD11b^+^ myeloid cells significantly increased in the spleen after DSS treatment and was restored to the normal level after antibiotic treatment. Other subtypes of myeloid cells, including granulocytes, monocytes, and macrophagocytes, showed similar patterns of improvement (Figure S2A). As shown in Figure 1(k), the infiltration of CD11b^+^ myeloid cells in the colonic lamina propria was much higher in the DSS group and was attenuated to the control group level after antibiotic treatment. Other subtypes of myeloid cells, including granulocytes, monocytes, and macrophagocytes, exhibited similar trends (Figure S2B). In addition, the proportion of Th17 cells was downregulated in the Neo group; however, there was no significant difference in the ratio of Tregs. In summary, all the antibiotics tested in the present study exhibited positive effects on alleviating susceptibility to DSS-induced colitis in female mice and reduced inflammatory cell infiltration in the colon.

### 3.2. Alleviating DSS-Induced Colitis in Male Mice with Different Narrow-Spectrum Antibiotics

**Figure 2:**
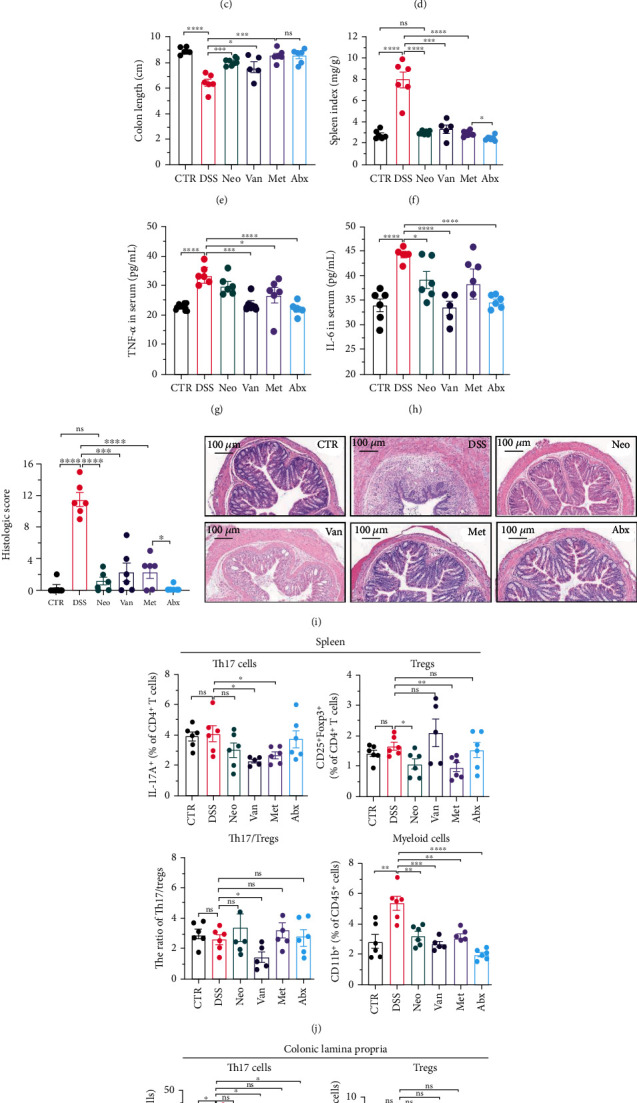
Effects of different antibiotics on DSS-induced colitis in male mice. (a) Study design: 6-week male C57BL/6 mice were given vancomycin, metronidazole, neomycin, antibiotic cocktail (mixture of vancomycin, metronidazole, neomycin and ampicillin), or regular water as control, respectively, for one week, and then challenged with 2% DSS (*n* = 6/group). (b) Body weight variation during the whole experiment process. (c) DAI score after DSS treatment. (d) Representative images and colon length (e) of the mouse colon. (f) Spleen index of different groups. Quantification of TNF-*α* (g) and IL-6 (h) in serum. (i) Histologic score and representative imaging of hematoxylin and eosin staining. (j) FACS analysis of immune cells in spleen including Th17 cells, Tregs, and myeloids. (k) FACS analysis of immune cell infiltration in colonic lamina propria including Th17 cells, Tregs, and myeloids. All data were presented as means ± SEM. ^∗^*P* < 0.05,  ^∗∗^*P* < 0.01, and^∗∗∗^*P* < 0.001.

An identical experimental design and protocol were applied to male mice, as shown in Figure 2(a). Again, the Neo and Met groups showed a slight decrease in body weight (Figure 2(b)), similar to that observed in female mice. The DAI scores in the Neo and Met groups were much lower than those in the DSS group (Figure 2(c)). Antibiotic treatment reversed the shortening of the colon (Figure 2(e)), splenic enlargement (Figure 2(f)), and intestinal congestion (Figure 2(d)), but not in the Van group. Moreover, downregulation of serum TNF-*α* and IL-6 levels was observed in all antibiotic treatment groups (Figures 2(g) and 2(h)). Histological analysis indicated that the DSS group exhibited the most severe morphological damage to the colon (Figure 2(i)). Apparent lymphocytic infiltration, crypt destruction, and epithelium appeared in the Van group, while intestinal epithelial structure remained intact in the Neo, Met, and Abx groups. Compared with the CTR group, DSS treatment did not affect the proportion of CD45^+^ and CD4^+^ T cells in the spleen, but antibiotic treatment upregulated these ratios (Figure S3A). As shown in Figure 2(j), there was a downward trend in Th17 cells in the spleen in the Van and Met groups. However, there was no significant difference in the ratio of Th17 to Tregs, except for the Van group. The proportion of myeloid cells increased in the spleen after DSS treatment, whereas antibiotics decreased this proportion to normal levels (Figure 2(j)), among which monocytes, a subtype of myeloid cells, decreased most significantly (Figure S3A). Here, antibiotics resulted in a marked increase in myeloid cell infiltration in the colonic lamina propria compared with that in the DSS group (Figure 2(k)), which was opposite that observed in female mice (Figure 1(k)). The same pattern was observed for several myeloid cell subtypes (Figure S3B). However, the proportion of Th17 cells and the ratio of Th17 cells to Tregs in the colonic lamina propria decreased significantly, which may reduce inflammation. In conclusion, antibiotics could also alleviate the development of colitis in male mice through mechanisms different from those in female mice, in which Neo and Met exhibited superior protection performance over Van and Abx.

### 3.3. Narrow-Spectrum Antibiotics Alleviated Colitis in the *α*PD-L1 Treatment of Murine Colon Cancer: Female Mice Group

**Figure 3:**
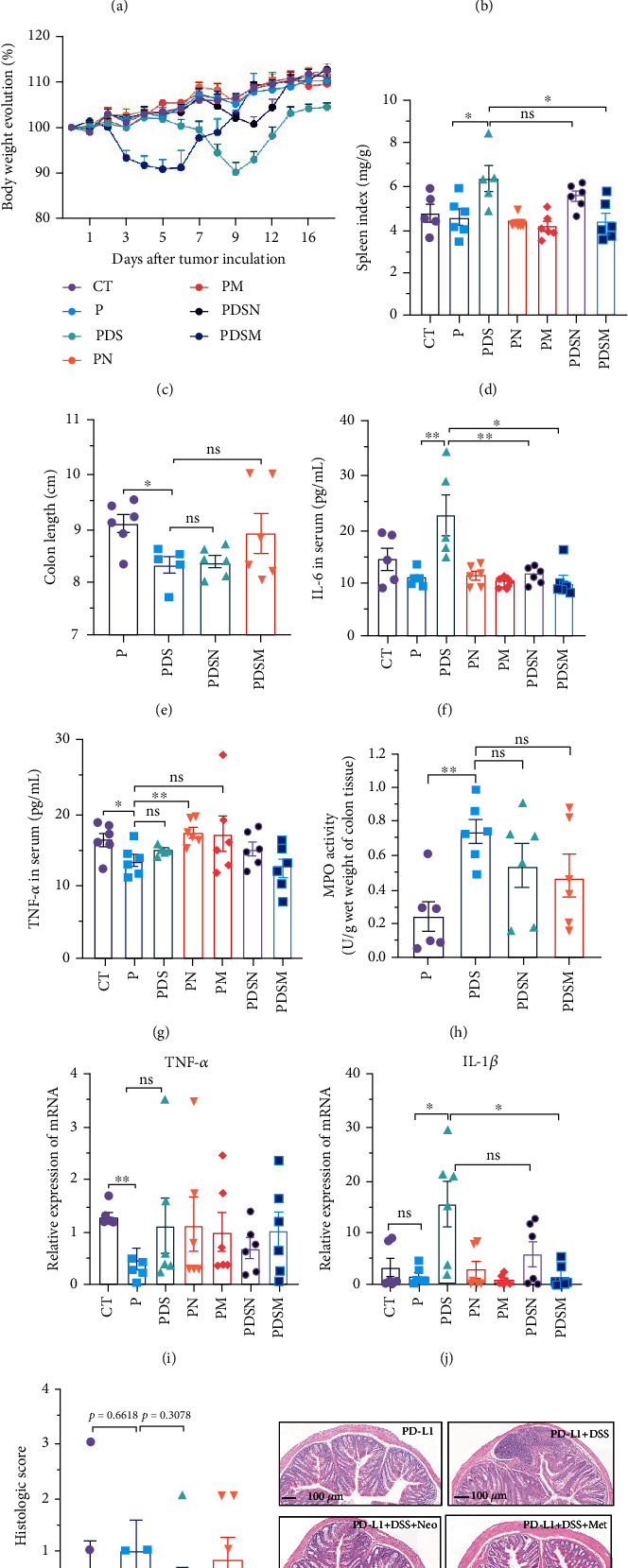
Neomycin and metronidazole slightly hindered the antitumor efficacy of anti-PD-L1 immunotherapy. (a) Study design: 6-week female C57BL/6 mice with DSS-induced colitis or without DSS were treated intraperitoneally (i.p.) with *α*PD-L1, either in combination with neomycin and metronidazole or without antibiotic. (b) Growth curve of tumor volume. (c) Body weight variation during the whole experiment process. (d) Spleen index of different groups. (e) Colon length of the mouse colon. (f) Quantification of IL-6 in serum. (g) Quantification of TNF-*α* in serum. (h) MPO activity of colon tissue. (i) Relative expression of mRNA about TNF-*α* in cecum tissue. (j) Relative expression of mRNA about IL-1*β* in cecum tissue. (k) Histologic score and representative imaging of hematoxylin and eosin staining. All data were presented as the means ± SEM. ^∗^*P* < 0.05,  ^∗∗^*P* < 0.01, and^∗∗∗^*P* < 0.001.

The effects of narrow antibiotics on the treatment of colitis associated with immunotherapy of murine colon cancer with *α*PD-L1 were first studied in female mice. Based on the above-mentioned results, Neo and Met were chosen for the following experiments because of their superior anti-inflammatory effects. The experimental design is shown in Figure 3(a), in which the acronyms are as follows: control (CT), *α*PD-L1(P), *α*PD-L1+DSS (PDS), *α*PD-L1+neomycin (PN), *α*PD-L1+metronidazole (PM), *α*PD-L1+DSS+neomycin (PDSN), and *α*PD-L1+DSS+metronidazole (PDSM). As shown in Figure 3(c), no significant body weight loss was observed in the PDSN and PDSM groups. Spleen enlargement was significantly attenuated in the PDSM group (Figure 3(d)), and colon shortening was alleviated to a certain extent, despite no statistical significance (Figure 3(e)). The serum levels of IL-6 were restored to normal in the antibiotic-treated groups (Figure 3(f)). Compared to the P group, there was an observable increase in the level of TNF-*α* in the PN and PM groups, which decreased after DSS treatment (Figure 3(g)). MPO is the predominant enzyme in neutrophil granulocytes.  Figure 3(h) shows that intestinal MPO activity markedly increased after DSS treatment and decreased in the antibiotic-treated groups. In addition, *α*PD-L1 reduced the mRNA level in the cecal mucosa compared with CTR, whereas the other groups showed no significant changes (Figure 3(i)). DSS treatment led to a drastic increase in the mRNA levels of IL-1*β* in the cecal mucosa, which was significantly decreased by Met treatment (Figure 3(j)). Two weeks after DSS modeling, the intestinal epithelial structure was not fully restored in the PDS group (Figure 3(k)). In contrast, a certain recovery in the intestinal histological injury was achieved in the PDSN group, although the difference was not significant. These results confirm the effectiveness of Neo and Met in alleviating colitis during *α*PD-L1 treatment in mouse colon cancer. However, it should be noted that the tumor volume in the late stages of Neo and Met treatment increased more rapidly than that in the *α*PD-L1 treated group (Figure 3(b)). This undesirable effect calls our attention to the effects of DSS on immunotherapy with *α*PD-L1.

**Figure 4:**
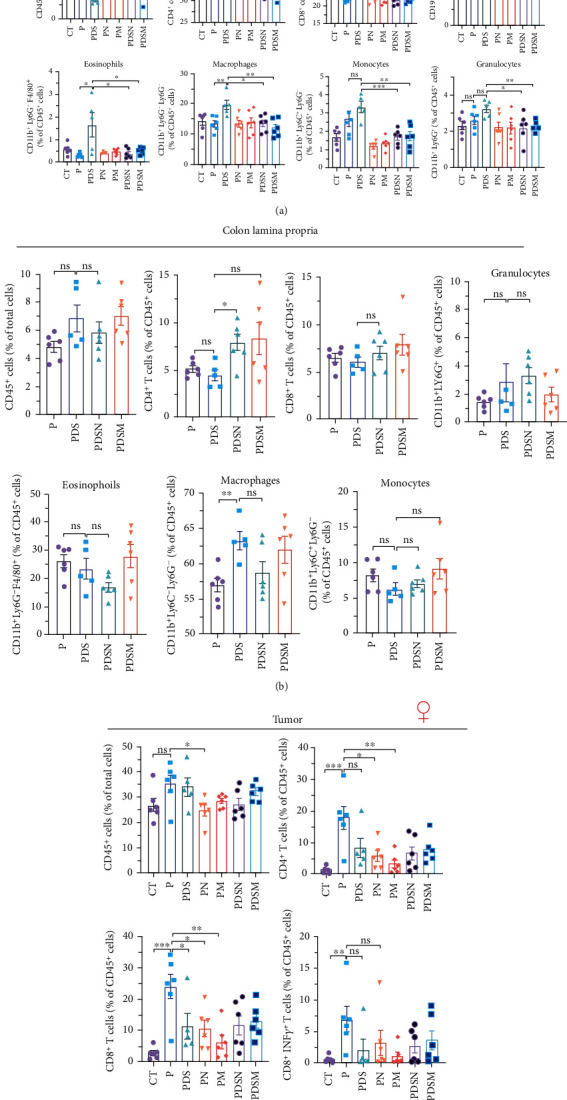
Antibiotics decrease CD11b^+^ cells in mesenteric lymph nodes (MLNs) and colonic lamina propria as well as the infiltration of CD8^+^ T cells in tumor microenvironment in female mice. (a) Immune cell variation in MLNs. (b) Inflammatory cell infiltration in colonic lamina propria. (c) Immune cell infiltration in tumor microenvironment. All data were presented as means ± SEM. ^∗^*P* < 0.05,  ^∗∗^*P* < 0.01, and^∗∗∗^*P* < 0.001.

Thus, we examined immune cell infiltration in the colon lamina propria, MLNs, and tumor microenvironment. MLNs are vital sites for maturation and differentiation of intestinal immune cells. As shown in Figure 4(a), although the proportions of CD45^+^ and CD4^+^ T cells in MLNs were reduced in the PDS group, the proportion of CD11b^+^ cells increased prominently, which was consistent with the results observed in Figures 1(j) and 1(k). In addition, antibiotics induced a significant reduction in CD11b^+^ myeloid cells in MLNs, such as eosinophils, macrophages, monocytes, and granulocytes. In contrast, the proportion of CD8^+^ T cells showed no significant changes. Notably, the ratio of CD19^+^ cells showed an apparent upward trend in the P and PDS groups, suggesting that B cells (CD19^+^) may also play a role in the tumor immune response and inflammation. No significant differences were observed in the infiltration of inflammatory cells, such as eosinophils, macrophages, monocytes, and granulocytes, in the colonic lamina propria (Figure 4(b)). Analysis of lymphocyte infiltration in the tumor microenvironment showed that both DSS and antibiotics reduced the proportion of CD8^+^ T cells and CD8^+^ INF*γ*^+^ T cells, which might account for the reduced antitumor activity in the other groups (Figure 4(c)). These results suggest that the palliative effect of Neo and Met on colitis is not affected when combined with *α*PD-L1 antibodies, but antibiotics slightly hindered the antitumor efficacy of immunotherapy in female mice at the late stage, as shown by the faster rate of tumor volume growth (Figure 4(b)) and reduced tumor infiltrating CD8^+^ T cells (Figure 4(c)).

### 3.4. Narrow-Spectrum Antibiotics Alleviated Colitis in the *α*PD-L1 Treatment of Murine Colon Cancer: Male Mice Group

**Figure 5:**
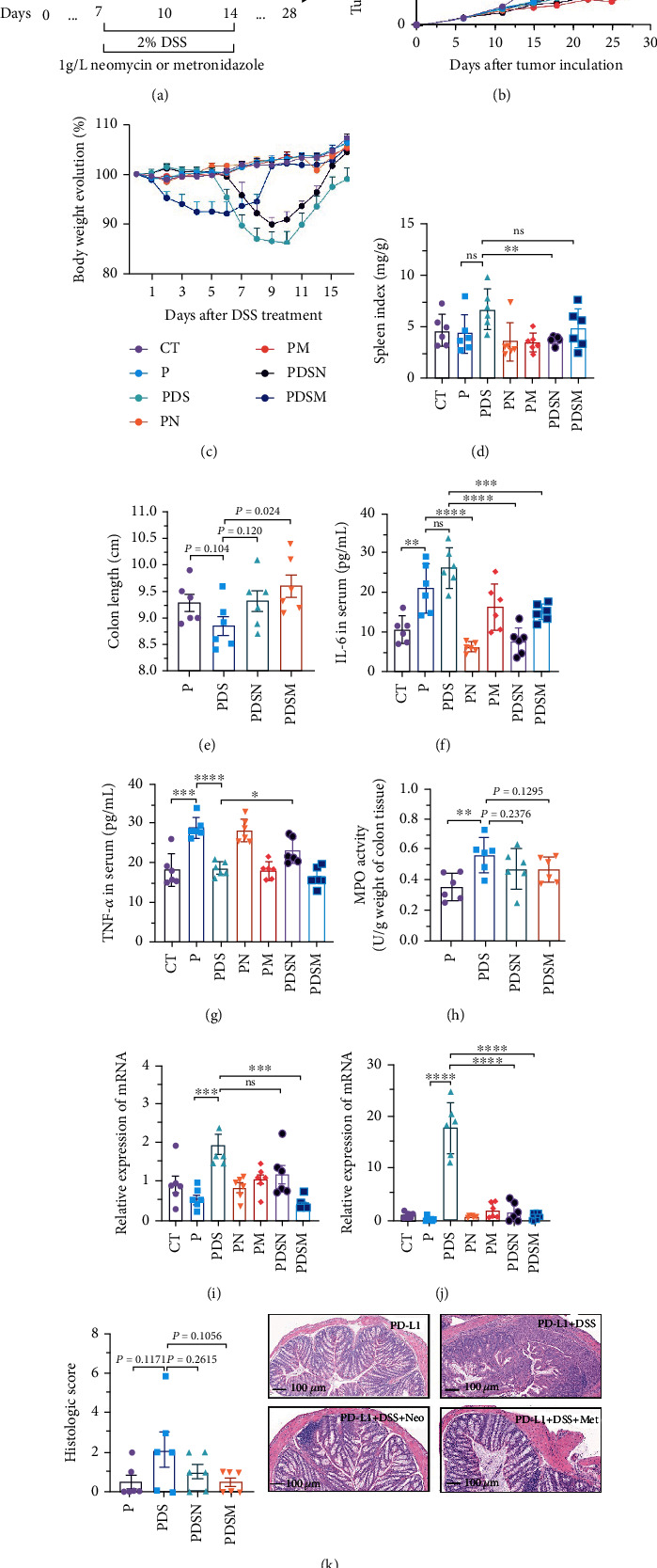
Neomycin and metronidazole did not hinder—and even enhanced the antitumor efficacy of combined anti-PD-1 immunotherapy in male mice. (a) Study design: 6-week male C57BL/6 mice with DSS-induced colitis or without DSS were treated intraperitoneally (i.p.) with *α*PD-L1, either in combination with neomycin and metronidazole or without antibiotic. (b) Growth curve of tumor volume. (c) Body weight variation during the whole experiment process. (d) Spleen index of different groups. (e) Colon length of the mouse colon. (f) Quantification of IL-6 in serum. (g) Quantification of TNF-*α* in serum. (h) MPO activity of colon tissue. (i) Relative expression of mRNA about TNF-*α* in cecum tissue. (j) Relative expression of mRNA about IL-1*β* in cecum tissue. (k) Histologic score and representative imaging of hematoxylin and eosin staining. All data were presented as means ± SEM. ^∗^*P* < 0.05,  ^∗∗^*P* < 0.01,  ^∗∗∗^*P* < 0.001, and^∗∗∗^*P* < 0.0001.

The animal protocol, which was the same as described above, is shown in Figure 5(a). As shown in Figure 5(b), the growth rate of the tumor volume in the PDS group was faster than that in the P group, similar to that observed in the female mouse group. It was noteworthy that application of both Neo and Met assisted the suppression of tumor growth, as compared with the P group (Figure 5(b)). This was different from that observed in female mice (Figure 4(b)). Both Neo and Met demonstrated a strong ability to alleviate colitis, as evidenced by reduced body weight loss in the PDSN and PDSM groups compared to the PDS group (Figure 5(c)). Neo and Met also showed strong protection against splenic enlargement and colon shortening (Figures 5(d) and 5(e)). Moreover, both antibiotics significantly reduced serum IL-6 levels with or without DSS treatment (Figure 5(f)). As for TNF-*α*, there was an upward tendency in the P group but a downward tendency in the PDS group (Figure 5(g)). This is different from the response of the female group shown in Figure 3(g). MPO activity was markedly elevated after DSS treatment and decreased in antibiotic-treated groups (Figure 5(h)), which was similar to the response of the female groups (Figure 3(h)). Moreover, DSS treatment led to a drastic increase in the mRNA levels of TNF-*α* and IL-1*β* in the cecal mucosa, which were significantly decreased by Neo and Met treatment (Figures 5(i) and 5(j)). Intestinal histological analysis also confirmed the protective effect of Neo and Met on the intestinal mucosa (Figure 5(k)), which was better than that observed in the female group (Figure 3(i)).

**Figure 6:**
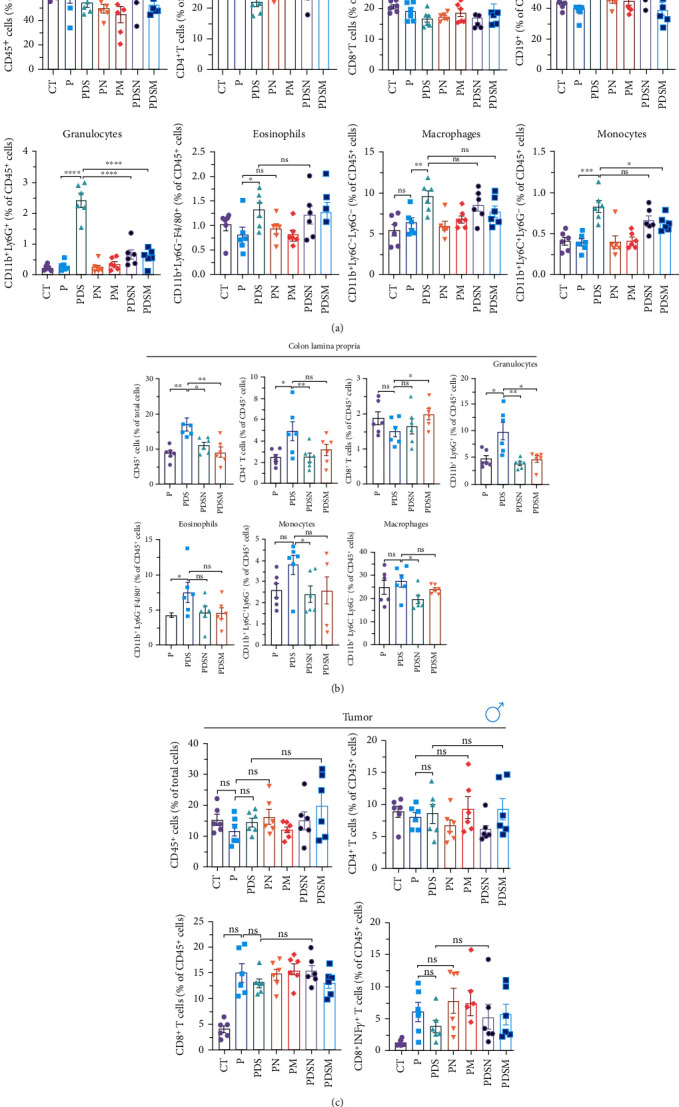
Antibiotics decrease CD11b^+^ cells in mesenteric lymph nodes (MLNs) and colonic lamina propria and increase the infiltration of CD8^+^ T cells in tumor microenvironment in male mice. (a) Immune cells variation in MLNs. (b) Inflammatory cell infiltration in colonic lamina propria. (c) Immune cell infiltration in tumor microenvironment. All data were presented as means ± SEM. ^∗^*P* < 0.05,  ^∗∗^*P* < 0.01, and^∗∗∗^*P* < 0.001.

We then examined the effects of these two antibiotics and *α*PD-L1 on immune cell infiltration in the colonic lamina propria, MLNs, and tumor microenvironment.  Figure 6(a) shows that there were no significant differences in the ratio of CD45^+^ cells among the groups in the MLNs. *α*PD-L1 treatment led to an increase in CD4^+^ T cells, while DSS and antibiotics resulted in a decrease in CD4^+^ T cells, which is similar to that observed in the female group. The proportion of CD11b^+^ cells dramatically increased after DSS treatment, whereas the application of antibiotics significantly suppressed the increase in CD11b^+^ cells in MLNs, especially granulocytes. The proportion of CD8^+^ T cells showed no obvious variation. Remarkably, the ratio of CD19^+^ cells increased in the PDS group and was restored to normal levels in the PDSN and PDSM groups, which is slightly different from the female group (Figure 5(a)).

Figure 6(b) shows that inflammatory cell infiltration in the colonic lamina propria was significantly higher in the PDS group than in the P group. Moreover, the proportion of CD11b^+^ cells, including eosinophils, macrophages, and monocytes, showed a sharp decrease. As shown in Figure 6(c), the application of colitis reduces the proportion of CD8^+^ T cells as well as CD8^+^ INF*γ*^+^ T cells, which may account for the reduced antitumor activity in the PDS group (Figure 6(b)). The application of Neo and Met slightly increased the proportion of CD8^+^ INF*γ*^+^ T cells, which is conducive to a better antitumor immune response.

### 3.5. The Connection of Colitis with Microbial Dysbiosis

**Figure 7:**
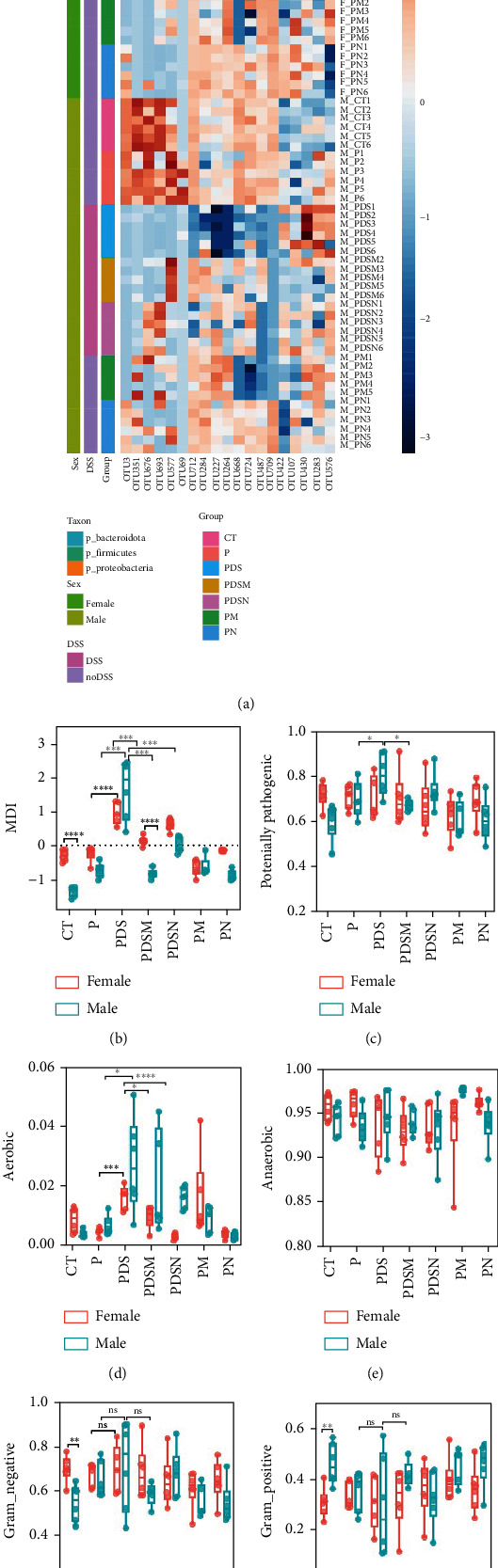
Microbial dysbiosis is associated with colitis. (a) Heatmap of dominated OTUs of colitis. (b) Microbial dysbiosis index (MDI) for each group. (c) BugBase predicted potentially pathogenic, aerobic (d), anaerobic (e), Gram-negative (f), and Gram-positive bacteria (g) in the microflora.

To understand the sexual dimorphism in response to the treatment of colitis with antibiotics during *α*PD-L1 immunotherapy, we examined the alteration of gut microbiota by 16S rRNA sequence analysis. We compared the discrepant OTUs in both male and female mice before and after DSS treatment, as shown in Figure 7(a). These discriminative OTUs are the dominant species in colitis and are used to calculate the microbial dysbiosis index (MDI) for each group as follows: log (total abundance of OTUs increased after DSS treatment/total abundance of OTUs decreased after DSS treatment). Compared with the *α*PD-L1 group (P), MDI increased significantly after DSS treatment in both male and female mice and decreased in the Neo and Met treatment groups (Figure 7(b)). Notably, MDI in male mice was better restored after antibiotic treatment than that in female mice. This may explain the better response to antibiotics found in the male group. Additionally, we performed phenotypic predictions using BugBase. As shown in Figure 7(c), DSS treatment resulted in a striking increase in potentially pathogenic bacteria, which was significantly reduced by Neo and Met. The aerobic bacteria showed a similar tendency (Figure 7(d)). No significant differences in anaerobic bacteria were observed in any of the groups (Figure 7(e)). However, DSS-induced inflammation led to a slight increase in Gram-negative bacteria, and Neo and Met strongly inhibited these bacteria (Figure 7(f)). Gram-positive bacteria showed the opposite trend (Figure 7(g)). Given these results, it can be concluded that colitis is strongly associated with microbial dysbiosis. Male mice appear to have a better therapeutic effect against microbial dysbiosis than female mice, which may partially account for the aforementioned sexual dimorphism.

### 3.6. The Effect of Gut Microbiota on Sexual Dimorphism in Colitis and Immunotherapy

**Figure 8:**
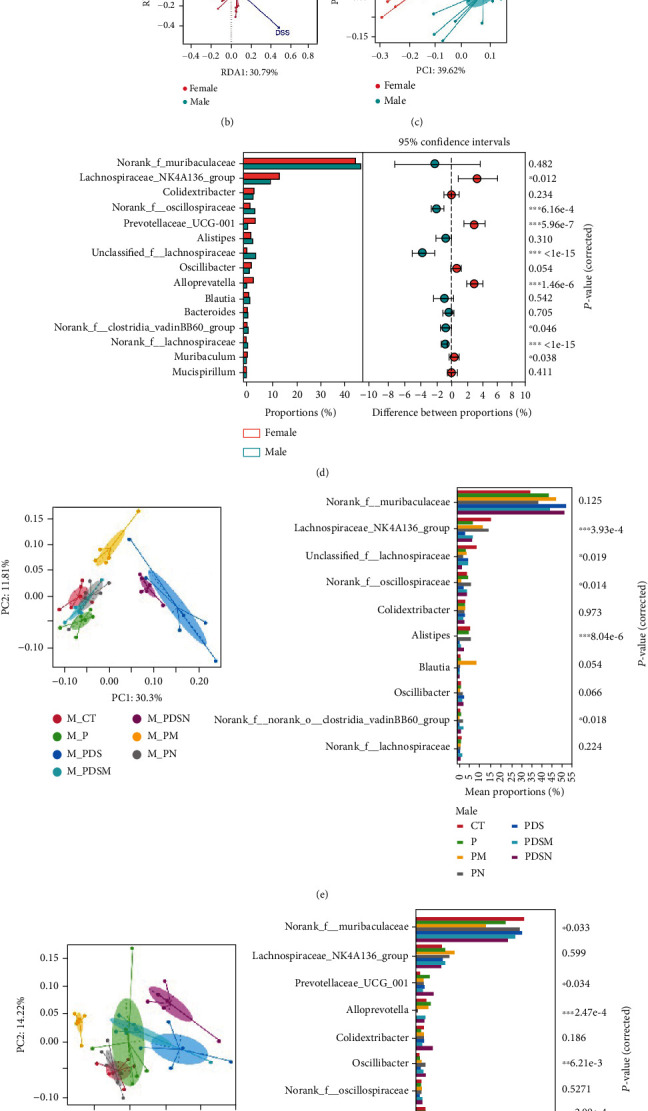
Alteration of the gut microbiota after *α*PD-L1 treatment. (a) Alpha-diversity of the gut microbiota. (b) Redundancy analysis (RDA). (c) Principal component analysis shows difference in the composition of the gut microbiota between male and female mice. (d) Wilcoxon rank-sum test bar plot on Genus level. (e) Principal component analysis and Kruskal-Wallis *H* test for male mice. (f) Principal component analysis and Kruskal-Wallis *H* test for female mice. All data were presented as means ± SEM. ^∗^*P* < 0.05,  ^∗∗^*P* < 0.01, and^∗∗∗^*P* < 0.001.

As shown in Figure 8(a), antibiotics and DSS led to a dramatic decrease in alpha diversity, including richness, chao1, and ACE, but Neo had less effect on the Shannon and Simpson indices. RDA indicated that sex had the greatest influence on gut microbiota, followed by DSS treatment (Figure 8(b)). PCA showed that there were significant differences in the microbiota composition between male and female mice (Figure 8(c)). Next, the analysis of the differential microorganisms at the genus level revealed that the abundance of *Lachnospiraceae_NK4A136_group* was meaningfully lower in male mice, whereas it was higher in female mice, and *Muribaculaceae* was the opposite (Figure 8(d)). PCA analysis and differential species identification in male mice are shown in Figure 8(e), in which the gut microbiota fluctuated considerably after Neo, Met, and DSS treatment in the male mouse group. The abundance of *Muribaculaceae* increased after *α*PD-L1 and DSS treatment. However, this trend was reversed in female mice (Figure 8(f)). *Muribaculaceae* have been shown to play an important role in azoxymethane/DSS-induced colorectal cancer, which may lead to diverse effects of antibiotics on colitis in male and female mice. *α*PD-L1 treatment significantly reduced the abundance of *Lachnospiraceae_NK4A136_group*, while Neo and Met increased it. In contrast, the abundance of *Lachnospiraceae_NK4A136_group* showed no significant differences in the female groups (Figure 8(f)). *Lachnospiraceae* has been confirmed to be associated with response to ICIs [35], which explains the disparate antitumor efficiency of antibiotics in male and female mouse groups. These results indicate that antibiotics have different effects on the abundance of some microorganisms in the gut and thus have a consequential impact on colitis and the therapeutic efficacy of *α*PD-L1.

## 4. Discussion

Although antibiotic treatment is effective in many kinds of bacterial infections and inflammation, it inevitably causes changes in the gut microbiota [36]. A large amount of clinical data has shown that the number and composition of intestinal flora in patients with colitis are notably altered [37]. Here, we found that all tested antibiotics could alleviate DSS-induced colitis to a certain degree. However, it is noted that although the antibiotic cocktail also showed a strong capability to relieve inflammation, the extensive reduction in gut microbiota caused a dramatic weight loss in mice from days 0-7, which was caused by impaired digestive function [38]. Hernández-Chirlaque et al. also reported that germ-free mice are more likely to develop DSS-induced colitis than mice treated with antibiotics [39]. Germ-free mice exhibited the lowest degree of inflammation, whereas the intestinal epithelium has been almost damaged. Although the degree of the inflammation in the antibiotic-treated group was stronger than that in germ-free mice, the intestinal epithelial barrier was better preserved, suggesting that complete elimination of the gut microbiota is not advisable. Therefore, narrow-spectrum antibiotics are preferred for colitis treatment.

Although antibiotics could alleviate DSS-induced colitis in both male and female mice, their effects on inflammatory cells were different. In the case of colitis in the context of *α*PD-L1 treatment of murine colon cancer, the relief of colitis by antibiotics was more pronounced in male mice, as evidenced by the lower histologic score (Figure 5(k)) and downregulated expression of mRNA of inflammatory cytokines (Figures 5(i) and 5(j)). Immune cell analysis of the spleen and colonic lamina propria also revealed differences between male and female mice. The ratio of CD11b^+^ myeloid cells in the spleen and colon lamina propria increased significantly after DSS treatment, while antibiotics decreased it to baseline in the female mouse group (Figures 1(j) and 1(k)). Shmuel et al. reported that the activation of intestinal macrophages and monocytes (two subtypes of CD11b^+^ myeloid cells) is the initial driver of inflammation [40]. Antibiotics reduce inflammation possibly by inhibiting the activation of these cells in female mice. The results of the immune cell analysis in male mice were slightly different from those in female mice. The percentage of CD11b^+^ myeloid cells in the colonic lamina propria increases remarkably after DSS treatment; this is further enhanced by the application of antibiotics (Figure 2(k)). In contrast, antibiotics significantly decrease the ratio of Th17 cells to Tregs in colonic lamina propria (Figure 2(k)), which is associated with the suppression of inflammation [41]. Collectively, these results suggest that colitis mitigation with antibiotics in male and female mice may be accomplished by regulating different types of inflammatory cells. Further investigation of the underlying mechanism is needed. Gut microbiota may influence colitis via sex hormones. Cornish and Ortizo confirmed that sex hormones are associated with exacerbation of clinical symptoms of inflammatory bowel disease (IBD) [42, 43]. Thus, the serum levels of estradiol and testosterone were determined by ELISA at the end of each experiment. As shown in Figure S4, testosterone levels were remarkably altered in the male colitis mice. DSS treatment reduced testosterone levels in male mice, which recovered to normal levels by antibiotic treatment, except with vancomycin. Such recovery was not observed in female mice. Markle et al. also revealed that FMT from male mice to female mice significantly altered the composition of the microbiota and increased testosterone levels, which finally alleviated islet inflammation [44]. This work showed that antibiotic treatment affected the host sex hormone levels, thereby having consequential effects on the regulation of gut immune cells, which merits further investigation.

Intestinal dysbiosis exacerbates colitis by promoting ubiquitination and accumulation of the innate immune adaptor stimulator of interferon genes (STING) in myeloid cells [40]. Olu et al. confirmed that oral administration of *Akkermansia muciniphila* strain BAA-835 considerably ameliorated colitis by modulating intestinal microecology [45]. Larsen et al. reported that lysozyme leveraged gut microbiota to inhibit DSS-induced colitis [46]. These findings indicate that restoring gut microbiota is conducive to alleviating colitis [47]. A recent clinical study has shown that the use of antibiotics, especially those with anaerobic activity, after ICI therapy is associated with an increased risk of severe ICI-mediated diarrhea and/or colitis; however, the antibiotic species and patient sex were not specified [48]. In the present study, we found that antibiotic treatment could promote the recovery of microbial communities, as evidenced by the regression of MDI, which is conducive to colitis therapy (Figure 7(b)). In addition, these antibiotics showed superior anti-inflammatory effects in male mice than in female mice. Furthermore, the proportion of Gram-negative bacteria was downregulated in the narrow-spectrum antibiotic treatment group. Furthermore, the proportion of Gram-negative bacteria was lower in the narrow-spectrum antibiotic treatment group, which may prevent STING accumulation in intestinal myeloid cells and alleviate intestinal inflammation [40]. Our findings demonstrate the potential to prevent colitis through the alleviation of microbial dysbiosis with antibiotics. Moreover, sex-dependent alterations in gut inflammation should be considered when treating intestinal inflammation with antibiotics.

The effects of gut microbes on the efficiency of immunotherapy with anti-PD-1/PD-L1 have been extensively reported [49–52]. Our previous study showed that the response efficiency of *α*PD-L1 was reinforced by colistin in male mice, whereas a detrimental effect of colistin was observed in female mice [25]. Clinical studies have also indicated that sex-related differences affect the response efficiency to ICIs. According to a metastudy, a lower the pooled overall survival hazard ratio was observed in male patients treated with ICIs compared with females [53]. However, when used in combination with chemotherapy, females derive more benefits from anti-PD-1/PD-L1 therapy than males [54]. The underlying mechanisms of sex-related differences in the antitumor efficiency of ICIs remain unknown. In the present study, we found that neomycin and metronidazole not only showed superior anti-inflammatory effects in both the male and female mouse groups but also improved the antitumor efficiency of *α*PD-L1 in the male mouse group. We also observed that the gut bacterial communities of male and female mice responded differently to treatment with DSS, antibiotics, and *α*PD-L1. Elderman et al. discussed the interactions between sexual dimorphism in immune responses and microbiota [55]. Ruff et al. reported that the immune system is partially shaped by gut microbiota [56]. Peng et al. showed that the abundance of *Lachnospiraceae* was associated with a better response to ICIs [30, 35]. In this study, both *α*PD-L1 and DSS treatment reduced the abundance of *Lachnospiraceae_NK4A136_group*, whereas neomycin and metronidazole prominently increased it in male mice (Figure 8(e)). However, these changes were not observed in female mice (Figure 8(f)). Byrd et al. pointed out that sex must be considered when designing microbiome-targeted therapies [57]. Based on these results, monitoring changes in gut microbiota may offer a route to probe sexual dimorphism in response to the treatment of colitis in the context of immunotherapy with *α*PD-L1.

In addition, the findings of the present study suggest that antibiotic treatment for colitis, especially ICI-associated colitis, needs to be tailored according to the type of cancer, type of antibiotic, treatment duration, and sex of the patient in clinical practice. Gordon et al. investigated the remission of antibiotics on colitis including 12 randomized controlled trials and found that a higher percentage of patients achieved clinical remission with antibiotics at 12 months [58]. Pinato et al. suggested that broad-spectrum antibiotics administered prior rather than concurrently to ICI therapy are associated with a worse treatment response and overall survival in patients with NSCLC and melanoma in routine clinical practice [59]. Unlike other oncological indications, a clinical study based on 450 ICI recipients with hepatocellular carcinoma by Fessas et al. revealed that antibiotics administered 30 days before or after ICI initiation were associated with improved benefit from immunotherapy [60]. Thus, more refined clinical trials specifically focusing on antibiotic treatment for ICI-associated colitis are warranted in the future.

## 5. Conclusions

In the present study, we tested the possibility of preventing the development of colitis using antibiotic treatment during anti-PD-L1 immunotherapy in mouse colon cancer. We found that narrow-spectrum antibiotics, such as neomycin and metronidazole, were effective in alleviating the symptoms of DSS-induced colitis in both male and female mice and appeared to have sex-dependent anti-inflammatory mechanisms. These antibiotics can attenuate the increased infiltration of granulocytes and myeloid cells in colon tissue induced by DSS in females while reducing the proportion of Th17 cells in males. Moreover, both Neo and Met can enhance the antitumor efficacy in colon cancer in male mice, while exhibiting destructive effects in female colon cancer, which may be attributed to the sex-biased responses of the gut microbiota. Collectively, these findings indicate that the application of narrow-spectrum antibiotics is promising for preventing the development of colitis during ICI therapy. The underlying mechanism of action of antibiotics in sex-dependent alterations in gut inflammation requires further investigation in the context of ICI treatment.

## Data Availability

The 16S rRNA sequencing dataset was deposited in the SRA database of NCBI with accession ID SUB10947980. All data that support the findings of this study are available upon reasonable request from the corresponding author.

## Supplementary Materials

Supplementary Materials Figure S1: gating strategy of immune cells. Figure S2: FACS analysis of immune cells in female mice: (A) FACS analysis of immune cells in spleen. (B) FACS analysis of immune cell infiltration in colonic lamina propria. All data were presented as means ± SEM. ^∗^*P* < 0.05,  ^∗∗^*P* < 0.01, and^∗∗∗^*P* < 0.001. Figure S3: FACS analysis of immune cells in male mice: (A) FACS analysis of immune cells in spleen. (B) FACS analysis of immune cell infiltration in colonic lamina propria. All data were presented as means ± SEM. ^∗^*P* < 0.05,  ^∗∗^*P* < 0.01, and^∗∗∗^*P* < 0.001. Figure S4: changes in sex hormone levels: The levels of estradiol (A) and testosterone (B) in female mice serum. The levels of estradiol (C) and testosterone (D) in male mice serum.
