# Supplementary material for: Sex-Biased Immune Responses to Antibiotics during Anti-PD-L1 Treatment in Mice with Colon Cancer
Source: J Immunol Res. 2022 Jul 19;2022:9202491. doi: 10.1155/2022/9202491 (PMC9325566; doi:10.1155/2022/9202491)
Supplement: Supplementary Materials — Figure S1: gating strategy of immune cells. Figure S2: FACS analysis of immune cells in female mice: (A) FACS analysis of immune cells in spleen. (B) FACS analysis of immune cell infiltration in colonic lamina propria. All data were presented as means ± SEM. ∗P < 0.05, ∗∗P < 0.01, and∗∗∗P < 0.001. Figure S3: FACS analysis of immune cells in male mice: (A) FACS analysis of immune cells in spleen. (B) FACS analysis of immune cell infiltration in colonic lamina propria. All data were presented as means ± SEM. ∗P < 0.05, ∗∗P < 0.01, and∗∗∗P < 0.001. Figure S4: changes in sex hormone levels: The levels of estradiol (A) and testosterone (B) in female mice serum. The levels of estradiol (C) and testosterone (D) in male mice serum. [file 9202491.f1.docx]

**Supplementary materials**


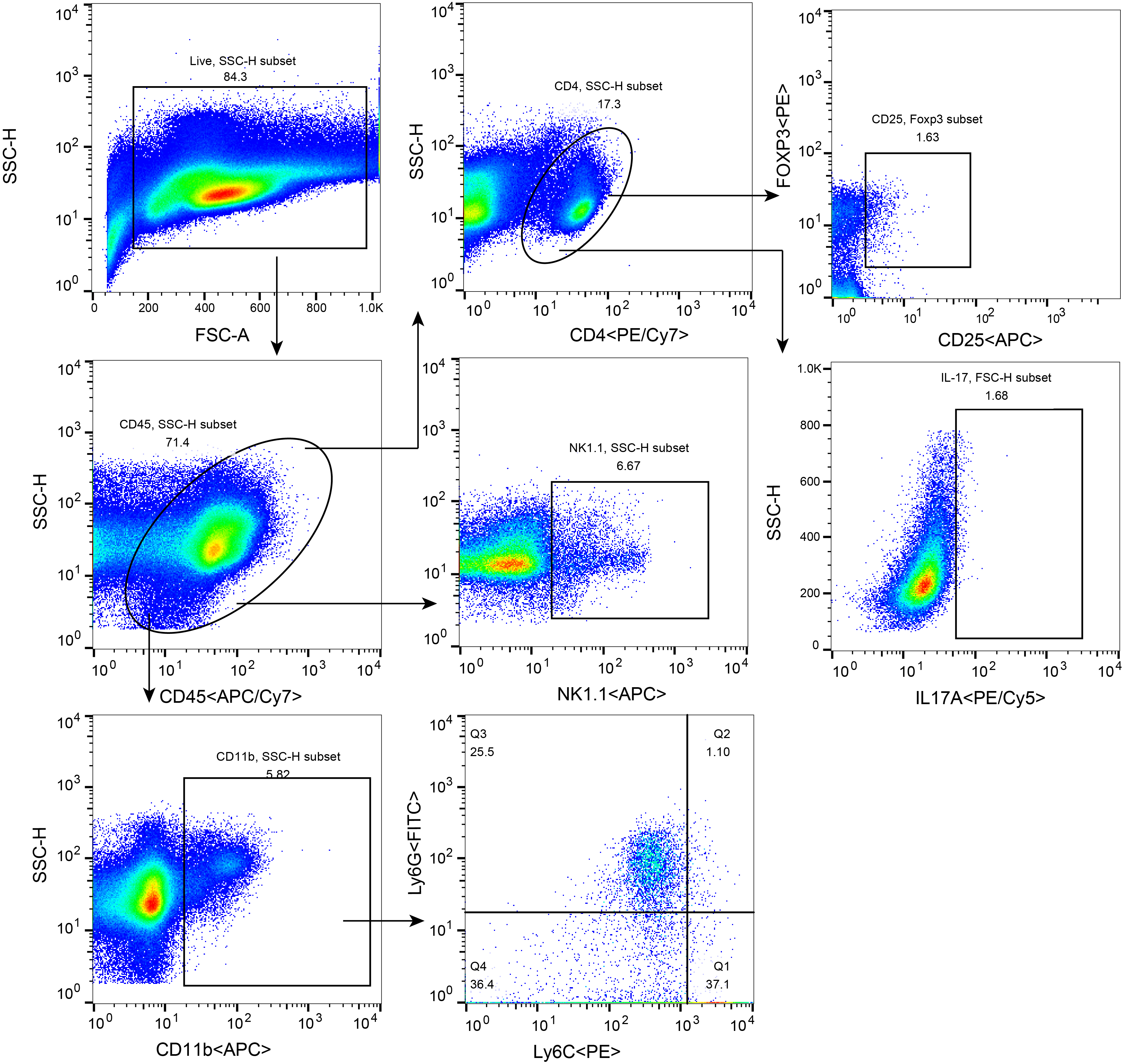


**Figure S1. Gating strategy of immune cells**


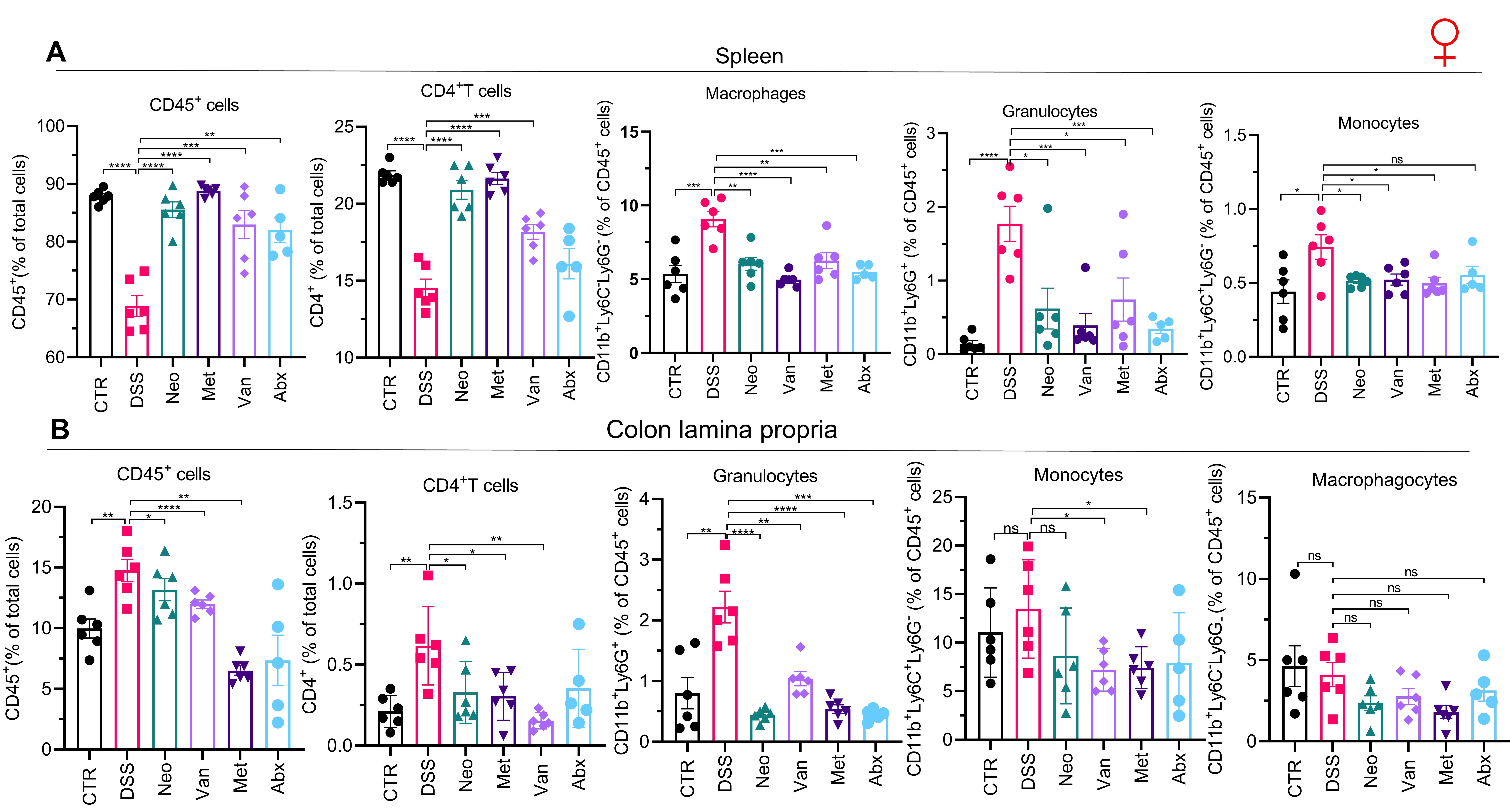


**Figure S2. FACS analysis of immune cells in female mice:** (A) FACS analysis of immune cells in spleen. (B) FACS analysis of immune cell infiltration in colonic lamina propria. All data were presented as means ± SEM. **P* <0 .05, ***P* < 0.01, ****P* < 0.001


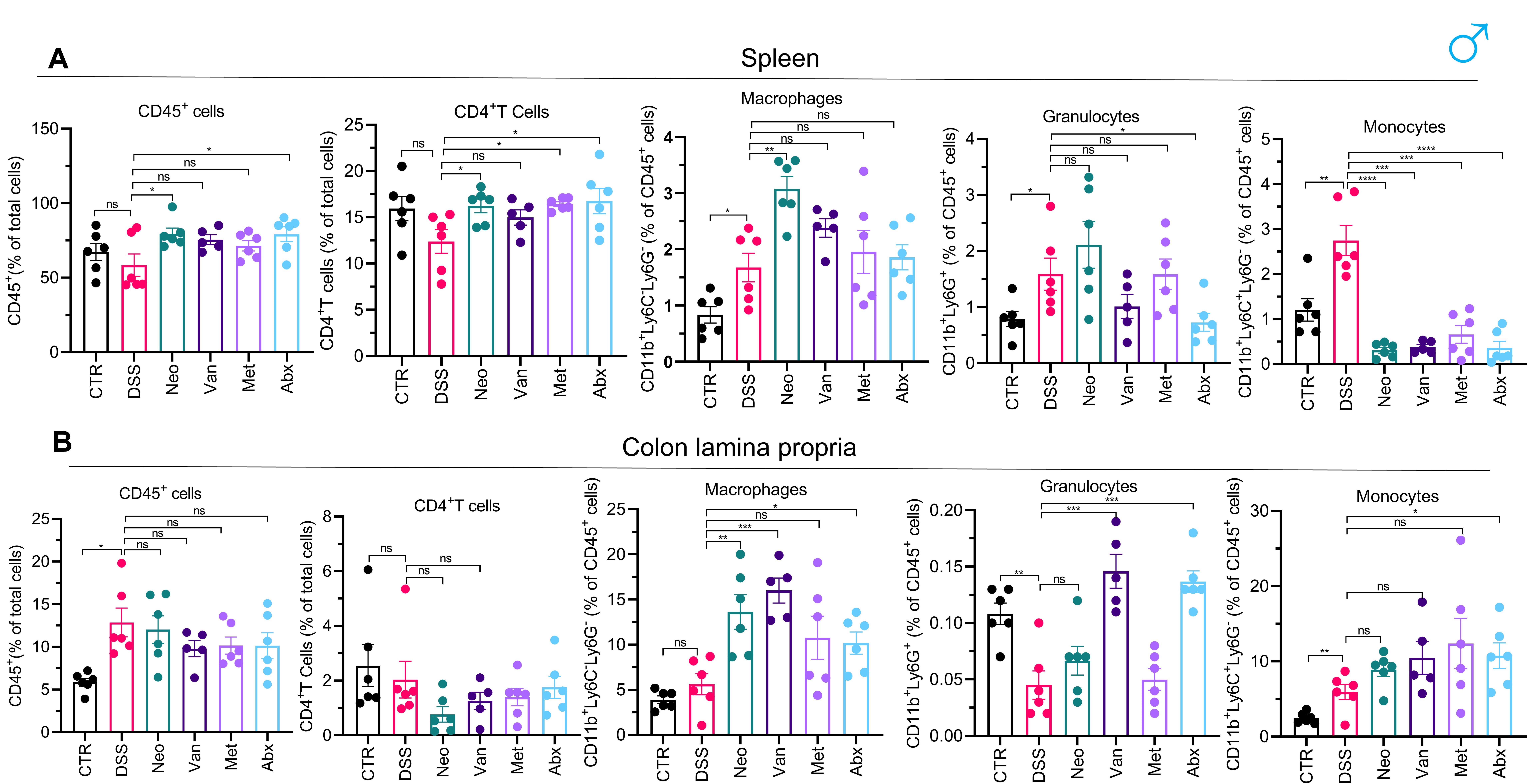


**Figure S3. FACS analysis of immune cells in male mice:** (A) FACS analysis of immune cells in spleen. (B) FACS analysis of immune cell infiltration in colonic lamina propria. All data were presented as means ± SEM. **P* <0 .05, ***P* < 0.01, ****P* < 0.001


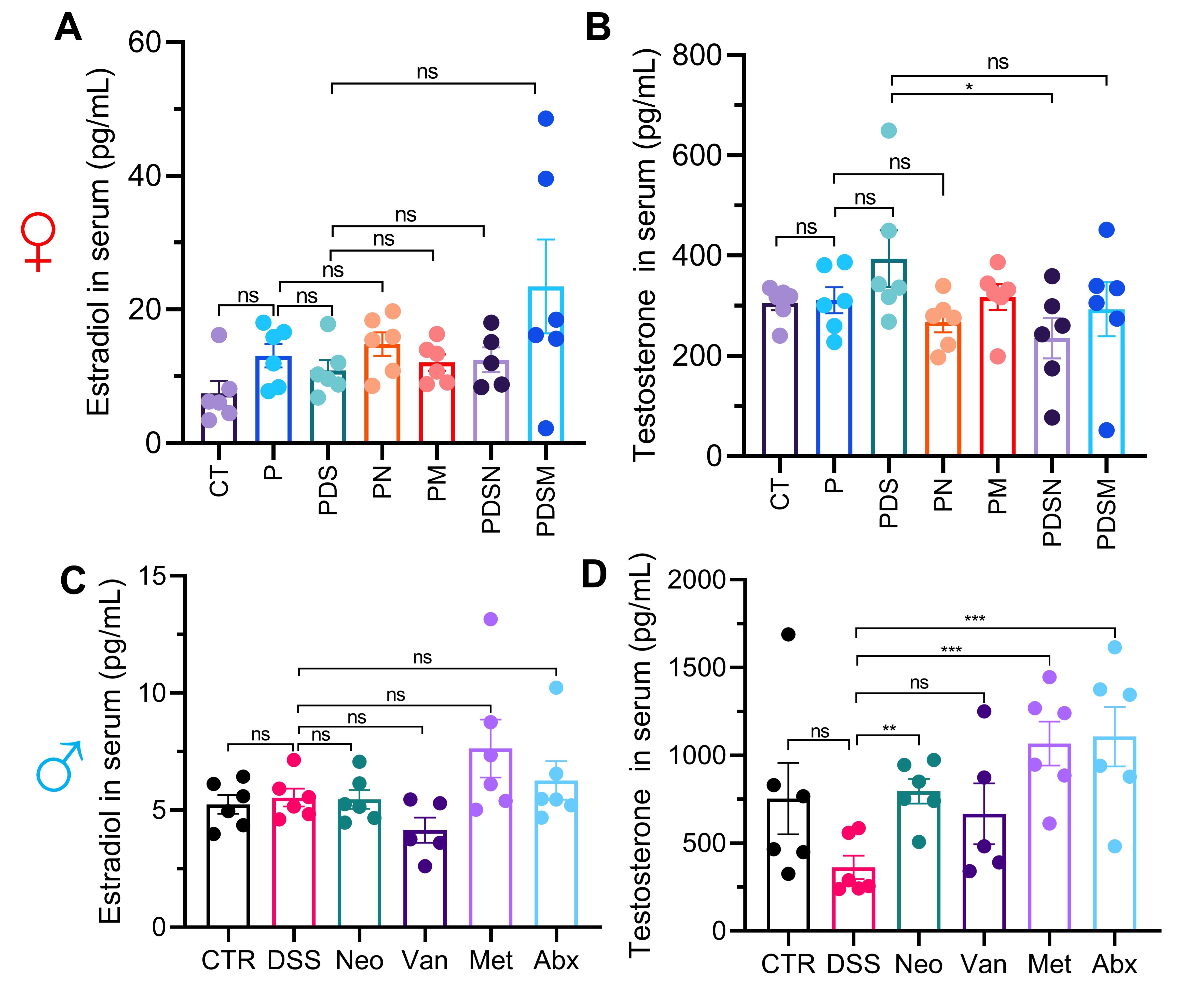


**Figure S4**. Changes in sex hormone levels: The levels of estradiol (A) and testosterone (B) in female mice serum. The levels of estradiol (C) and testosterone (D) in male mice serum.
